# A nurse-led clinic for patients consulting with osteoarthritis in general practice: development and impact of training in a cluster randomised controlled trial

**DOI:** 10.1186/s12875-016-0568-y

**Published:** 2016-12-21

**Authors:** Emma L. Healey, Chris J. Main, Sarah Ryan, Gretl A. McHugh, Mark Porcheret, Andrew G. Finney, Andrew Morden, Krysia S. Dziedzic

**Affiliations:** 1 Arthritis Research UK Primary Care Centre, Research Institute for Primary Care & Health Sciences, Keele University, Staffordshire, UK; 2 Staffordshire and Stoke on Trent Partnership NHS Trust, Haywood Hospital, Stoke-on-Trent, UK; 3 Keele University, School of Nursing and Midwifery, Clinical Education Centre, University Hospital of North Staffordshire, Stoke-on-Trent, UK; 4 School of Healthcare, University of Leeds, Yorkshire, UK; 5 School of Social and Community Medicine, University of Bristol, Gloucestershire, UK

**Keywords:** Osteoarthritis, Practice nurses, Training, Primary care, Implementation, Evaluation

## Abstract

**Background:**

Despite a lack of service provision for people with osteoarthritis (OA), each year 1 in 5 of the general population consults a GP about a musculoskeletal condition such as OA. Consequently this may provide an opportunity for practice nurses to take an active role in helping patients manage their condition. A nurse led clinic for supporting patients with OA was developed for the MOSAICS study investigating how to implement the NICE 2014 OA Guideline core recommendations. This paper has two main objectives, firstly to provide an overview of the nurse-led OA clinic, and secondly to describe the development, key learning objectives, content and impact of the training to support its delivery.

**Methods:**

A training programme was developed and delivered to provide practice nurses with the knowledge and skill set needed to run the nurse-led OA clinic. The impact of the training programme on knowledge, confidence and OA management was evaluated using case report forms and pre and post training questionnaires.

**Results:**

The pre-training questionnaire identified a gap between what practice nurses feel they can do and what they should be doing in line with NICE OA guidelines. Evaluation of the training suggests that it enabled practice nurses to feel more knowledgeable and confident in supporting patients to manage their OA and this was reflected in the clinical management patients received in the nurse-led OA clinics.

**Conclusions:**

A significant gap between what is recommended and what practice nurses feel they can currently provide in terms of OA management was evident. The development of a nurse training programme goes some way to develop a system in primary care for delivering the core recommendations by NICE.

**Trial registration:**

The cluster trial linked to this training was conducted from May 2012 through February 2014 by the Arthritis Research UK Primary Care Centre, Keele University, UK (Trial registration number ISRCTN06984617).

**Electronic supplementary material:**

The online version of this article (doi:10.1186/s12875-016-0568-y) contains supplementary material, which is available to authorized users.

## Background

Osteoarthritis (OA) is a major public health issue due to its impact on increasing numbers of older adults [1, 2]. Arthritis Care [3] determined that 8.5 million adults in the UK had pain and disability attributed to OA, and by 2020 OA has been predicted to be the 4th largest cause of disability and the 6th leading cause of years lived with disability [4]. Between 1990 and 2010, disability due to OA in the UK increased by 16% [5]. It has also been estimated that by 2030 over half the UK population will be over the age of 50 and nearly the same proportion will be obese; leading to an estimate of 17 million people in the UK living with OA [3]. In the UK over a seven-year period about a third of older adults consult their general practitioner (GP) with OA [6], but despite this OA management is not seen as a high priority for general practice [7] and both patients and health care professionals (HCPs) generally concur that OA should receive more attention and better consistency of care [8].

Self-management of chronic long-term conditions (LTCs) and professional support for self-management are emphasized in National Health Service (NHS) policy [9]. In 2008, the National Institute for Health and Care Excellence (NICE) first produced a guideline for the care and management of OA and recommended that all patients with OA should be offered three core treatments when they first present: education and access to information, advice on local muscle strengthening exercise and general aerobic fitness, and if appropriate advice on losing weight [10]. Paracetamol and/or topical nonsteroidal anti-inflammatory preparations were also recommended as first-line analgesics [10]. This guideline was subsequently updated in 2014 [11] and the NICE Quality Standards for OA were produced the following year [12], both of which recommend core treatments as a priority for implementation.

**Fig. 1:**
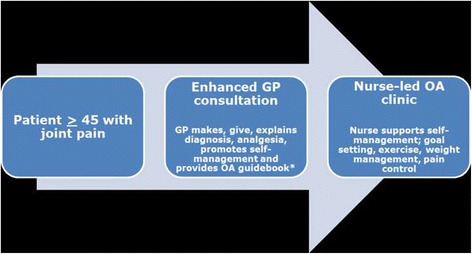
The model OA consultation. *Grime and Dudley (2011) (weblink:http://www.keele.ac.uk/media/keeleuniversity/ri/primarycare/pdfs/OA_Guidebook.pdf)

Despite such clinical guidelines, there is a gap between the care that is recommended for OA and the care that patients receive [7, 13, 14]. Previous studies have also shown that among patients with knee OA, core treatments are equally self and doctor initiated [14, 15]. In a recently undertaken study (the MOSAICS (Management of OSteoArthritis In ConsultationS) study) [16] an intervention for supporting patients with OA to use these core treatments was developed in the form of a model OA consultation. The Whole Systems Informing Self-Management Engagement (WISE) model [17] was adopted as the guiding framework for the model OA consultation which comprises three components: i) an OA Guidebook developed with user involvement to provide patient-centred and evidence based information (http://www.arthritisresearchuk.org/arthritis-information/keele-oa-guide.aspx) [18], ii) delivery of an enhanced OA consultation by a GP [16, 19] (assessment of the presenting problem, and, if OA diagnosed, the diagnosis given and explained, patient expectations addressed, and referral to a nurse-led OA clinic) and iii) a practice based nurse-led OA clinic in which the patient was offered up to four appointments with a nurse specifically trained to support OA self-management. The development of the model OA consultation is described elsewhere [16]. Figure 1 demonstrates where the nurse-led OA clinic sits within the model OA consultation.

### Rationale for a nurse-led OA clinic and a training programme to support its delivery

In the UK practice nurse’s work within general practice as a key part of the primary health care team, where they plan and provide nursing care, treatment and health education to patients of all ages. Over recent years the role of practice nurses has extended within primary care, with more responsibility for the management of LTCs [20]. Several factors have led to this expansion, including issues related to cost, the need to increase provision of care to improve access, the availability of doctors, and the under-utilised skills and expertise of nurses [21]. Currently in the UK, the majority of patients with OA are managed in primary care by their GP. Practices nurses working alongside GPs in general practice have the opportunity to offer the core treatments recommended in the NICE OA guidelines as well as referring to other members of the multidisciplinary team when further intervention such as physiotherapy is required, and foster a team based approach by recommending referral. While nurses working in general practice have experience of reviewing patients with a range of LTCs, these reviews are predominantly for patients with conditions linked to the NHS Quality and Outcome Framework, such as diabetes and asthma. Only recently, the need for nurse training and education to develop competence, confidence and knowledge regarding the management of musculoskeletal problems, has been demonstrated [22]. Lillie et al. [23] examined the educational needs of nurses caring for people with arthritis and concluded that future training programmes on OA management should provide nurses with the opportunity to develop knowledge and skills in providing advice on exercise and pain medication.

In the absence of established OA training for nurses, a training programme needed to be developed specifically to provide practice nurses with the appropriate skill set and knowledge to confidently deliver the nurse-led OA clinic in the MOSAICS study [24]. This paper has two main objectives, firstly to provide an overview of the nurse-led OA clinic, and secondly to describe the development, key learning objectives, content and impact of the training to support its delivery.

## Methods

### The nurse-led OA clinic

**Fig. 2:**
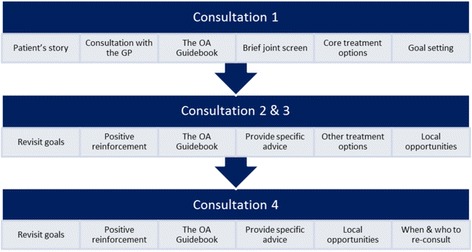
Content and overview of the nurse-led component of the model OA consultation

The nurse-led OA clinic is a component of a model OA consultation developed to be tested in terms of clinical and cost effectiveness in the MOSAICS randomised controlled trial. Full details of this trial can be found in the trial protocol [16]. In brief, eight general practices participated in the trial and were randomly allocated to two clusters: ‘intervention’ or ‘control’. The nurse-led OA clinic was set up and implemented by nurses working in one of the 4 practices recruited to the MOSAICS trial who were randomised to receive the training and deliver the intervention. Those patients aged 45 years and over that consulted with joint pain during the 6 month recruitment period were eligible for this trial. The aim of the nurse-led OA clinic was to support patients to increase their use of the NICE OA core treatments and first-line analgesia in OA self-management [10, 11]. A patient-centred approach was adopted to support OA self-management [25] within the OA clinic and included: goal setting; addressing the need for pain relief; encouraging opportunities for increasing physical activity; identifying the need for local muscle strengthening exercises, and demonstrating exercises; where appropriate encouraging referral for weight management; joint examination if needed; and the use of the OA Guidebook as appropriate (see Fig. 2).

The nurse-led OA clinic was delivered by nurses working in the practices which were randomised to deliver the intervention, and who had attended the OA training programme. Each patient referred to the clinic was offered up to four appointments over a 3-month period and it was up to both the nurse and patient to agree how many appointments were required. The first appointment of up to 30 min, enabled an assessment of the patient’s needs and preferences, with subsequent appointments lasting up to 20 min. The nurse-led clinic was supported by an OA computer template to record care, case report forms (CRFs) and research processes undertaken at each appointment [13] and a toolkit of resources was available for the practice nurse to use and included pedometers, exercise and pain diaries for patient completion, goal setting sheets, Arthritis Research UK information leaflets (e.g. Osteoarthritis, Keep Moving) and links to local weight management and physical activity services, all of which were offered to patients as appropriate.

### Development of the training programme

A draft 4-day training programme was developed by members of the MOSAICS research team which addressed the perceived learning needs of practice nurses who would deliver the OA clinic in the study. To help determine the content of the training programme the research team drew on the findings of a scoping exercise of pertinent literature to identify similar or relevant training packages. The ‘challenging arthritis’ program provided by the voluntary organisation Arthritis Care [26], the ‘Arthritis Self-Management Program’ [27], the NHS Health trainer Handbook [28] and chronic disease self-management programmes such as the Expert Patient Program [29] were all examined for content. In-house training from OA trials within our research centre (e.g. SMOoTH (hand OA) [30], BEEP (knee pain) [31]) were also examined.

A four-step approach was taken to refining the draft training programme to develop the final content and style of the training programme:

1. A Patient and Public Involvement (PPI) discussion group to provide patient views on the draft training programme
2. A Practice Nurse Advisory Group (PNAG) meeting to provide professional views on the draft training programme
3. A pilot test of the training programme with feedback from trainees and trainers
4. A training development group to review the findings from steps 1 to 3 and make recommendations for the content and style of the final training programme

1. Patient and Public Involvement (PPI) discussion group
  A group of patients and members of the public (PPI) with OA (*n* = 7) were invited to attend a meeting to introduce the concept of the nurse led clinic and obtain their perspectives on the proposed content of the training programme.
  The PPI group were particularly keen to ensure the nurses were able to provide a clear explanation to patients of their OA diagnosis and the potential side effects of the medications frequently prescribed. They suggested that the nurses should use the content of the OA Guidebook, promote the use of pain and exercise diaries by patients and conduct joint assessments. The group also felt a wider knowledge of the treatments recommended by NICE for OA, rather than just focussing on the core treatments, was important.
2. Practice Nurse Advisory Group (PNAG) meeting
  An evening meeting of the PNAG, which was made up of local practice nurses and nurse practitioners (*n* = 7, all female), was held to obtain their perspectives on the proposed content and format of the nurse-led OA clinic, and to comment on whether the proposed training programme would equip nurses with necessary knowledge and skills to deliver the OA clinic.
  The key components that the nurses thought important to cover in the training programme included: joint familiarisation, characteristics of OA pain and understanding the impact of OA on the individual, an overview of the anatomy and physiology of OA and the NICE OA Guideline, where to signpost patients to self-management opportunities within the community linked to local and voluntary initiatives/schemes (e.g. local walking groups, weight watchers), when to refer patients to other NHS based health care professionals (e.g. physiotherapy, podiatry), and how to demonstrate and provide advice on strengthening and aerobic exercise.
3. Pilot test of the training programme
  The draft training programme was revised, to take account of the views of the PPI group and the PNAG, and piloted between September and October 2011 with a group of four nurses (two research nurses, one practice nurse and one experienced rheumatology nurse) who were recruited via convenience sampling.
  The pilot test of the training was evaluated by the trainers and trainees through trainer reflections on each section of the programme, “real-time” observations and suggestions from trainers and trainees and formal daily evaluation by trainees. Whilst it was felt that the overall content and timescale were satisfactory, it was agreed that changes should be made to the balance of the programme and mode of delivery, in line with learning theory and practice [32]. The suggested changes to the training programme are shown in Table 1.

**Table 1:** Suggestions for change included in the final training programme

1. Increase the opportunities for group discussion by reducing the amount of formal (didactic) teaching in sessions and move a considerable amount of material from face to face sessions into resources to be used on a personal basis: written papers, PowerPoint slides on CD or other media.
2. Use an experienced clinical facilitator to lead and co-ordinate the programme, with expert contributions that could consist of a short didactic presentation with group discussion as a major component.
3. Explore and build on the trainees’ clinical expertise from the start, supplementing this with specific knowledge about the nature of OA, its management and the role of self-management.
5. Integrate practical application and skills development throughout the programme, particularly around the use of the OA guidebook and the OA toolkit.
6. Dedicate one full day to developing OA specific knowledge and skills with simulated patients, ensuring adequate preparation for this component and allowing sufficient time to develop and reflect on these skills and their application to real practice, particularly with regard to returning patients and helping to address any difficulties which the patients had encountered in putting their plans into practice
4. Training development group
  The group consisted of nine individuals with various backgrounds (general practice, research, education, social science, exercise science, nursing, physiotherapy, rheumatology and clinical psychology). The specific role of the group was to review and critically appraise the findings from steps 1 to 3, and feed these into the development of nurse training programme.

### Evaluation of the final training programme

Prior to the training a baseline questionnaire was sent to all the nurses based at the practices involved in the MOSAICS trial (*n* = 25) (see Additional file 1). Characteristics including number of years qualified, current role, and any relevant training or experience (e.g. musculoskeletal training, orthopaedic placements) were collected. A modified version of the Practitioner Self-Confidence scale (score range 4–20, greater scores indicate greater confidence levels [33]) and answers to a number of questions regarding knowledge of OA management (assessed using a 5-point Likert scale) were also collected at baseline.

In order to evaluate the impact of the training on confidence and knowledge, the nurses from practices randomised to deliver the intervention and who went on to attend the finalised training programme (*n* = 9) were re-sent the same questionnaire post training to determine any changes in their knowledge and confidence to facilitate self-management for people consulting with OA.

The extent to which the training impacted clinically on the management of those seen in the OA clinic and whether practice nurses drew on the key elements of optimal OA management identified in the training was determined by examining individual patients CRFs which were completed by the practice nurses for all patients attending the OA clinics set up for the MOSAICS trial.

To gain some initial feedback from the nurses that attended the training, and obtain specific evaluation of the individual days and sessions, all 9 participants were also asked to complete an evaluation form at the end of each training day and to score each session from 1 (extremely unsatisfactory) to 5 (extremely satisfactory).

## Results

### Baseline characteristics of the practice nurses involved in the MOSAICS trial

Of those invited to complete the baseline questionnaire, 21 (84%) nurses responded. The responders had been qualified for a mean of 25.8 years (sd 10.8) and 2 (9.5%) were further qualified as nurse practitioners. In terms of relevant experience or training, 5 (23.8%) reported having experience or training in Orthopaedics and 7 (33.3%) in Rheumatology. Only 1 nurse reported that she had heard of or read the NICE OA guidelines. On average, the nurses reported very low confidence (16.1 sd 3.0) regarding the decisions needed when caring for patients with chronic joint problems, and 14 (66.6%) reported having no confidence in examining joints.

### Content and delivery of the final training programme

The training was delivered to the nurses from the practices randomised to deliver the intervention (*n* = 9) on a full day (9 am–4.30 pm) once a week for 4 weeks (see Table 2 for the key learning objectives). This allowed the nurses to reflect on the training and provide the opportunity to put the knowledge and skills learnt into practice in between training days. The full content of the training programme is presented in Additional file 2.

**Table 2:** Key learning objectives of the nurse training programme

1. Demonstrate an understanding of what it means to be ‘patient centred’ when helping to support patient self- management for OA.
2. Demonstrate an understanding of the impact of different communication styles between the nurse & the patient on enabling patients to have an active role in self-management for OA.
3. Demonstrate an understanding of different perspectives of self-management for OA including clinical, psychological, policy & social perspectives.
4. Demonstrate expertise in supporting the patient through the process of goal setting.
5. Demonstrate an understanding of the physical, psychological & social impact of OA.
6. Demonstrate an understanding of pharmacological & non- pharmacological methods of pain management.
7. Demonstrate an understanding of the role of information, exercise and weight management in the management of OA.

The training took place at Keele University and was primarily delivered by members of the training development group. The training programme was supported by a training manual and the toolkit. Homework tasks were set prior to and during the training, whereby all trainees were asked to read some key literature [17, 34–36] and to familiarise themselves with the contents of the training manual, toolkit and the OA Guidebook. This material was discussed and referred to throughout the training. The methods of training delivery included a mixture of didactic teaching, small group discussion, practical sessions, and role play with simulated patients of the initial and follow-up appointments.

Formal trainer-led didactic sessions were kept to a minimum during the training. However, where this was deemed necessary, the sessions were made as interactive as possible and complemented by handouts and group discussion. Key topics addressed in these discussions included the ‘what’ and ‘how’ of the nurse-led consultation, the style of the consultation and communication skills.

Two types of experiential learning were used: (1) role play; and (2) carrying out a consultation with a simulated patient.

1. Role play
  This was normally conducted in pairs with one of the pair taking on the role of the practice nurse while the other took on that of the patient. This method was mostly employed during the joint familiarisation, goal setting and exercise/physical activity sessions where the nurses were respectively expected to: familiarise themselves with the joints of the hand, knee, hip and foot; practise the use of the SMART (specific, measurable, attainable, realistic, and time-bound) tool for agreeing goals with the patients [37]; and practise demonstrating joint specific exercises.
2. Consultations with simulated patients
  This method of consultation skills training was informed by previous literature which utilised context-bound training to change clinical behaviour [38–40]. A simulated patient is a person who takes on the role of a patient and is trained to present specific symptoms and hold particular beliefs and attitudes that are relevant to the objectives of the training. The scenario for a simulated patient consists of the problem which the patient is to present with, their past medical and social history, their ideas, concerns and expectations about the problem and, for this scenario, their knowledge and beliefs about OA and its treatment.
  In this training programme the simulated patients were used in the group teaching sessions to allow the trainees to practise consultation skills and receive feedback from other trainees and the trainers. The simulated patient scenarios were developed to reflect the issues which the practice nurses would most likely face when delivering an OA consultation. A list of ideas, beliefs, attitudes, expectations about these issues which the Training development group had encountered in clinical practice and/or in qualitative research interviews [41] was agreed. The list was debated, expanded and modified and a final list of issues to be considered in developing the simulated patient scenario was drawn up. On reviewing the list it was decided that three basic scenarios should be developed, each one covering a different aspect of self-management: exercise, weight loss and pain management. It was also decided that each scenario would have two versions with different presenting symptoms: one with chronic knee pain and one with chronic hip pain (see Additional file 3).

### Evaluation of the final training programme

In terms of the specific evaluation of the individual days and sessions, all 9 participants completed evaluation forms at the end of each training day. Overall, the nurses were very positive about the training, they all appeared engaged and satisfied with the training, (with all but one participant, who scored all sessions as 3,) scoring all sessions 4 or 5 out of 5.

**Fig. 3:**
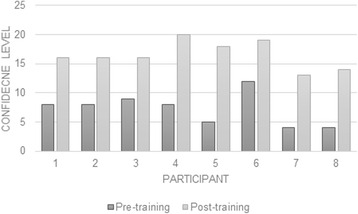
Pre and post training self-confidence in diagnosing and managing chronic joint problems (greater scores indicate greater confidence, modified from Smucker et al. 1998)
(ii) Clinical management of OA
  Over the study period, 268 patients were seen by the trained practice nurses in an OA clinic. With regards to the uptake of the core treatments, at the first consultation the nurses reported that they discussed the OA guidebook with the majority of patients (*n* = 254, 94.8%). Joint specific exercises and general physical activity were also advised and discussed with 89.6 and 88.1% of patients, respectively. Weight management was discussed with 74.6% of patients. By the fourth consultation the use of the OA guidebook reduced significantly and was only used with approximately 30% of those that attended a fourth consultation (*n* = 53). This might reflect the nurses becoming more confident in their OA knowledge and consultation skills and not needing to refer to the guidebook so often. Exercise, physical activity and weight management remained a consistent focus during each of the four consultations.

(i) Knowledge and confidence regarding the management of OA
  Eight (88.8%) out of the nine nurses that attended the training completed the post-training questionnaire. The results suggested that that training increased knowledge of OA, with all nurses reporting that they had heard of or had read the NICE OA guidelines. Prior to the training the nurses felt ‘partly informed’ about the causes, prognosis, burden, the range of treatments, what patients with OA can do to manage their condition and what nurses can do to support patients with OA, with scores ranging from 2.9 (sd 0.8) to 3.4 (sd 0.7) out of 5. However, after the training all scores focused on knowledge improved, with the most noticeable differences found in knowledge of the range of treatments (4.8 sd 0.5) and what patients with OA can do to manage their condition (4.8 sd 0.5), with the majority of participants now feeling ‘very well informed’.
  Confidence also improved markedly in those that were trained with scores improving by 9.2 points on average, shifting from an average of 7.3 to 16.5 out of 20 on the scale in these individuals, with greater scores indicating greater confidence (see Fig. 3). Interestingly, there was a large shift following the training in the percentage of nurses feeling OA management was part of their role within primary care (4.7 to 62.5%).

## Discussion

This paper provides the rationale for the nurse-led OA clinic, including the model OA consultation and the associated training programme, and describes the development, content and impact of the training programme which was devised to enable nurses to encourage a patient centred approach to increase the uptake of the NICE OA core treatments [10, 11], and was tested in the MOSAICS trial [16].

In line with previous research, the data gathered from the baseline questionnaire indicated a significant gap between the NICE OA guidance and what the nurses involved in this study felt they could currently provide in terms of OA management. Therefore, despite nurses now taking a leading role in the management of LTCs such as diabetes and asthma [42], there was a clear lack of knowledge and confidence regarding the provision of self-management support for patients consulting in general practice for OA.

The changes suggested from the pilot training programme were well-received by the trainees and improved the flow of the training. The post-training evaluations appear to demonstrate that the nurses were engaged with the training and that it enabled them to be more knowledgeable and confident in offering positive messages about what OA is and supporting the use of the core treatments. This seems to be reflected in the clinical management, as it appears that the nurses focused on the core treatments of information provision, encouraging weight management and physical activity. This finding was also reflected in the post intervention group interview with the nurses, a component of the qualitative work within the MOSAICS study [43, 44]. In a group interview the nurses described how they felt more confident dealing with patients who consulted with OA and were able to modify their relationship with OA patients, because they had the opportunity to play more of an active role in patient care [43]. Ong et al. [44] also reported that the nurses recognised that they had lacked skills and knowledge of OA previously, felt that the training had helped to up skill them in this regard, and left them feeling they could manage patients with OA better than previously, and boosted their professional standing. Despite the nurses being encouraged to use the OA guidebook as part of each consultation, its use dropped off considerably over the course of the four consultations This may be due to the nurses feeling more knowledgeable and confident as reflected in the findings from the group interview, clinical management and post-training questionnaire data, and therefore they felt less reliant on the use of the written information.

The main limitation of the data is that it is self-reported from a small sample of nurses. While there are limitations in terms of the generalisability of this data and there may be reporting bias, it does suggest that the nurses were promoting the uptake of the core recommendations in the NICE guidelines, which was the aim of the training.

It is felt that the training programme could be delivered and implemented internationally as the NICE OA guideline includes international research. It is a relatively low cost programme (no expensive kit needed) as the main financial outlay is the cost of the trainers and attending the programme for the trainees. It is envisaged that the trainers could train and support other trainers in the rest of the UK and across Europe to deliver this model. In other countries, where there may not be practice nurses, this model would also be relevant for all HCPs involved in the care of people with OA and could be supported by others such as physiotherapists. However, is it important to recognise that it may be difficult for HCPs to have 4 days out of clinical practice for training, especially for a non-QoF long term condition. In line with this our team have been collaborating with Education for Health and Arthritis Research UK to refine the training into a shorter face-to-face package complemented by an online resource that may more attractive to the NHS. This package is now being implemented in the JIGSAW implementation project [45].

## Conclusions

This study demonstrates a significant gap between what is recommended and what practice nurses feel they are currently equipped to provide in terms of OA management. The development of a practice nurse training programme goes some way to develop a system in primary care for delivering the core NICE recommendations. This training programme will enable practice nurses to deliver the nurse-led component of the model OA consultation which is to be tested in the MOSAICS trial [16].

## Additional files

Additional file 1: Pre training questionnaire. (DOC 492 kb)
Additional file 2: Final practice nurse training outline. (DOCX 25 kb)
Additional file 3: The different patient scenarios used in the training. (DOCX 11 kb)

## Abbreviations

CRF: Case report form
GP: General practitioner
HCP: Health care professional
LTC: Long-term condition
MOSAICS: Managing Osteoarthritis in Consultations
NHS: National Health Service
NICE: National Institute for Health and Care Excellence
OA: Osteoarthritis
PNAG: Practice Nurse Advisory Group
PPI: Patient and Public Involvement
QOF: Quality outcomes framework
SMART: Specific, measurable, attainable, realistic, time
WISE: Whole Systems Informing Self-Management Engagement.

## References

[1] Jordan KP, Kadam UT, Hayward R et al. Annual consultation prevalence of regional musculoskeletal problems in primary care: an observational study. BMC Musculoskeletal disorders 2010; 144: http://www.biomedcentral.com/1471-2474/11/144. 10.1186/1471-2474-11-144 PMC2903510 20598124

[2] Jinks C, Jordan K, Croft P (2006). Disabling knee pain--another consequence of obesity: results from a prospective cohort study. BMC Public Health.

[3] Arthritis Care. OA Nation Report. 2012. https://issuu.com/arthritiscare/docs/oa_nation_2012_report.

[4] Woolf AD, Pfleger B (2003). Burden of major musculoskeletal conditions. Bull World Health Organ.

[5] Murray C, Richards MA, Newton JN (2013). UK health performance: findings of the Global Burden of Disease Study 2010. Lancet.

[6] Jordan KP, Joud A, Bergknut C (2014). International comparisons of the consultation prevalence of musculoskeletal conditions using population-based healthcare data from England and Sweden. Ann Rheum Dis.

[7] Steel N, Bachmann M, Maisey S (2008). Self-reported receipt of care consistent with 32 Quality Indicators: national population survey of adults aged 50 or more in England. BMJ.

[8] Mann C, Gooberman-Hill R (2011). Health care provision for osteoarthritis: Concordance between what patients would like and what health professionals think they should have. Arthritis Care Res.

[9] Goodwin N, Curry N, Naylor C, Ross S, Duldig W (2010). Managing people with long-term conditions.

[10] NICE (2008). OA: The care and management of adults with OA. National Institute of Health and Clinical Excellence.

[11] NICE (2014). OA: The care and management of adults with OA. National Institute of Health and Clinical Excellence.

[12] Osteoarthritis NICE quality standard [QS87] Published date: June 2015. https://www.nice.org.uk/guidance/qs87/chapter/list-of-quality-statements. Accessed 12 May 2016.

[13] Edwards JJ, Jordan KP, Peat G (2015). Quality of care for OA: the effect of a point-of-care consultation recording template. Rheumatology.

[14] Porcheret M, Jordan K, Jinks C, Croft P, Primary Care Rheumatology Society (2007). Primary care treatment of knee pain--a survey in older adults. Rheumatology.

[15] Jinks C, Ong BN, Richardson J (2007). A mixed methods study to investigate needs assessment for knee pain and disability: population and individual perspectives. BMC Musculoskelet Disord.

[16] Dziedzic KS, Healey EL, Pushpa-Rajah A (2014). Implementing the NICE OA guidelines in Primary Care: The Management of OSteoArthritis In ConsultationS (MOSAICS) study protocol. Implement Sci.

[17] Kennedy AP, Rogers AE, Bower P (2007). Support for self care for patients with chronic disease. BMJ.

[18] Grime J, Dudley B. Developing written information on OA for patients: facilitating user involvement by exposure to qualitative research. Health Expect. 2011; doi: 10.1111/j.1369-7625.2011.00741. 10.1111/j.1369-7625.2011.00741.x PMC5060719 22070445

[19] Porcheret M, Main CJ, Croft PR (2014). Development of a behavior change intervention: a case study on the practical application of theory. Implement Sci.

[20] MacDonald W, Rogers A, Blakeman T, Bower P (2008). Practice nurses and the facilitation of self-management in primary care. J Adv Nurs.

[21] Horrocks S, Andreson E, Salisbury C (2002). Systematic review of whether nurse practitioners working in primary care can provide equivalent care to doctors. BMJ.

[22] Fletcher MJ, Oliver S, Cook A, Albrow HA (2012). An investigation into practice nurses’ need for further education in musculoskeletal care. Pract Nurs.

[23] Lillie K, Ryan S, Adams J (2013). The educational needs of nurses and allied healthcare professionals caring for people with arthritis: results from a cross-sectional survey. Musculoskeletal Care.

[24] Dziedzic KS, Healey EL, Main CJ (2013). Implementing the NICE Osteoarthritis Guidelines in Primary Care: A Role for Practice Nurses. Musculoskeletal Care.

[25] Little P, Everitt H, Williamson I (2001). Preferences of patients for patient centred approach to consultation in primary care: observational study. BMJ.

[26] Buszewicz M, Rait G, Griffin M (2006). Self management of arthritis in primary care: randomised controlled trial. BMJ.

[27] Lorig K, Mazonson P, Holman HR (1993). Evidence suggesting that health education for self-management in patients with chronic arthritis has sustained health benefits while reducing health care costs. Arthritis Rheum.

[28] Michie S, Rumsey N, Fussell N, et al. Improving health: Changing behavior, NHS Health trainer Handbook. Department of Health. 2008. www.healthcheck.nhs.uk/document.php?o=621

[29] Department of Health (2001). The expert patient: a new approach to chronic disease management in the 21st century.

[30] Dziedzic K, Nicholls E, Hill S (2015). Self-management approaches for osteoarthritis in the hand: a 2 × 2 factorial randomised trial. Ann Rheum Dis.

[31] Foster NE, Healey EL, Holden MA (2014). Improving the effectiveness of exercise for knee pain in older adults in primary care: The BEEP trial protocol. BMC Musculoskelet Disord.

[32] Merriam SB. Andragogy and Self-Directed Learning: Pillars of Adult Learning Theory New Directions for Adult and Continuing Education. 2001;89:3–14. http://umsl.edu/~wilmarthp/modla-links-2011/Merriam_pillars%20of%20anrdagogy.pdf.

[33] Smucker DR, Konrad TR, Curtis P, Carey TS (1998). Practitioner self-confidence and patient outcomes in acute low back pain. Arch Fam Med.

[34] Main CJ, Buchbinder R, Porcheret M, Foster N (2010). Addressing patient beliefs and expectations in the consultation. Best Pract Res Clin Rheumatol.

[35] Morden A, Jinks C, Ong BN (2011). Lay models of self-management: how do people manage knee osteoarthritis in context?. Chronic Illness.

[36] Nelson ME, Rejeski J, Blair SN (2007). Physical activity and public health in older adults: recommendation from the American College of Sports Medicine and the American Heart Association. Med Sci Sport Exercise.

[37] Schut HA, Stam HJ (1994). Goals in rehabilitation teamwork. Disabil Rehabil.

[38] Cals JW, Scheppers NA, Hopstaken RM (2007). Evidence based management of acute bronchitis; sustained competence of enhanced communication skills acquisition in general practice. Patient Educ Couns.

[39] Lane C, Rollnick S. The use of simulated patients and role-play in communication skills training: A review of the literature to August 2005. Patient Education and Counseling. 2007;67(1-2):6713–20. http://www.sciencedirect.com/science/article/pii/S0738399107000912. 10.1016/j.pec.2007.02.011 17493780

[40] Rollnick S, Kinnersley P, Butler C (2002). Context-bound communication skills training: development of a new method. Med Educ.

[41] Holden MA, Nicholls EE, Young J, Hay EM, Foster NE (2012). The role of exercise for knee pain: what do older adults in the community think?. Arthritis Care Res.

[42] Laurant M, Reeves D, Hermens R (2005). Substitution of doctors by nurses in primary care. Cochrane Database Syst Rev.

[43] Morden A, Brooks L, Jinks C (2015). Research “push”, long term-change, and general practice. J Health Organ Manag.

[44] Ong BN, Morden A, Brook L (2014). Changing policy and practice: Making sense of national guidelines for osteoarthritis. Soc Sci Med.

[45] Hay EM, Dziedzic KS, Foster NE, et al. Clinical osteoarthritis and joint pain in older people: optimal management in primary care. Programme Grants for Applied Research, in press.

