# Supplementary material for: A nurse-led clinic for patients consulting with osteoarthritis in general practice: development and impact of training in a cluster randomised controlled trial
Source: BMC Fam Pract. 2016 Dec 21;17:173. doi: 10.1186/s12875-016-0568-y (PMC5178095; doi:10.1186/s12875-016-0568-y)
Supplement: Additional file 1: — Pre training questionnaire. (DOC 492 kb) [file 12875_2016_568_MOESM1_ESM.doc]

Appendix 1.


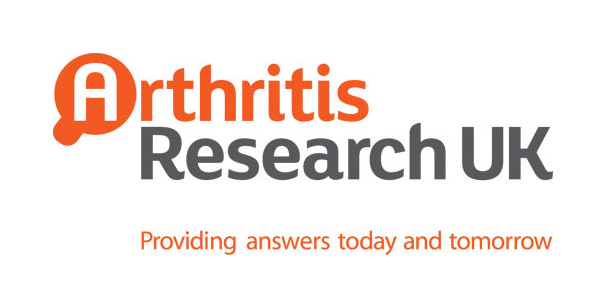


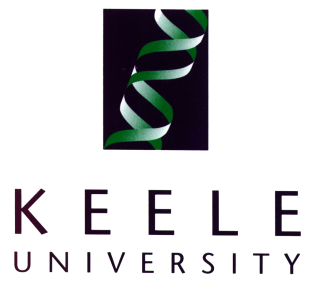


# Management of OSteoArthritis In ConsultationS study

# (MOSAICS)

## Arthritis Research UK Primary Care Centre

## Nurse practitioner/ Practice nurse

## Training Evaluation Questionnaire 1

**LREC Number:** Version 1 19/5/2011

IRAS Project code:

## Managing OSteoArthritis In ConsultationS

# (MOSAICS Study)

## Instructions for this questionnaire

**Please answer all the questions**

**X**

**The questions can be answered by putting a cross in the box like this**

**or by circling a number like this 3 4 5 6**

**Please write in BLOCK CAPITALS where appropriate**

**When you have finished please check that you have answered all of the questions and then return the questionnaire in the envelope enclosed. You do not need a stamp.**

**Please return the questionnaire in the next two weeks.**

**The answers you give in the questionnaire will be treated in the strictest confidence.**

**If you have any queries please contact June Handy on 01782 733964**

## **Thank you again for your help with this research study.**

| Section 1 - About you |
| --- |

|  | What year did you qualify as a nurse? | | | | | |  | |  | |  |  |
| --- | --- | --- | --- | --- | --- | --- | --- | --- | --- | --- | --- | --- |
|  |  | | | | | | | | |  | | |
|  | In what capacity are you a nurse at your practice? | | | | | | | | |  | | |
|  | Practice nurse | |  | Nurse practitioner | | | | | |  | | |
|  |  | |  |  | | | | | |  | | |
|  | Are you working | | | | | | | | | | | |
|  | Full time (30 hrs or more) | |  | Part time (30 hrs or less) | | | | | |  | | |
|  |  | |  |  | | | | | |  | | |
|  | Have you received any specific training in the field of musculoskeletal medicine since you qualified as a nurse? | | | | | | | | | | | |
|  | Yes...... | |  | If yes please give details | | No….. | | | |  | | |
| ........................................................................................................................................., | | | | | | | | | | | | |
| ......................................................................................................................................... | | | | | | | | | | | | |
|  | Have you ever worked in the field of Rheumatology or Orthopaedics? | | | | | | | | | | | |
|  | Yes..... | |  | If yes please give details | | No…. | | | |  | | |
| ......................................................................................................................................... | | | | | | | | | | | | |
| ......................................................................................................................................... | | | | | | | | | | | | |
|  | Are you? Male…… | |  | Or Female……. | | | | | |  | | |
|  | Do you have, or have ever suffered from, joint problems? | | | | | | | | | | | |
|  | Yes..... | |  |  | | No….. | | | |  | | |
|  |  |  | | |  | | |  | | | | |

| Section 2 - Your views about chronic joint problems |
| --- |

##### INTRODUCTION - IMPORTANT PLEASE READ

##### We are seeking the views of nurses participating in the MOSAICS Study about chronic joint problems.

##### By chronic joint problems we mean joint pain and associated symptoms that have been present for more than 3 months, and that are most likely attributable to osteoarthritis.

##### We do not mean problems resulting from:

##### A fracture

##### Infection

##### Inflammatory arthritis

##### Gout

##### Metastasis

##### Following surgery

| The following 4 statements are about the decisions you make when caring for patients with chronic joint problems. Please answer these questions by putting a cross in the one box in each row which best describes your answer. | | | | | | |
| --- | --- | --- | --- | --- | --- | --- |
|  | | | | | | |
|  |  | **Strongly**  **disagree** | **Disagree** | **Not sure** | **Agree** | **Strongly agree** |
|  | I lack the diagnostic tools or knowledge needed to effectively assess patients with chronic joint problems |  |  |  |  |  |
|  | I know exactly what to do to effectively treat patients with chronic joint problems |  |  |  |  |  |
|  | I am very comfortable treating patients with chronic joint problems |  |  |  |  |  |
|  | I am well prepared to manage chronic joint problems |  |  |  |  |  |

| **Below is a list of possible causes for a patient developing chronic joint problems. Please indicate the extent to which you agree or disagree with these causes by putting a cross in one box for each row.** | | | | | | |  |
| --- | --- | --- | --- | --- | --- | --- | --- |
|  |  | Strongly disagree | Disagree | Neither agree or disagree | Agree | Strongly agree | |
|  | Hereditary/runs in the family |  |  |  |  |  | |
|  | Being overweight/obese |  |  |  |  |  | |
|  | A person’s own mental attitude e.g. thinking about life negatively |  |  |  |  |  | |
|  | A person’s emotional state e.g. feeling down, anxious |  |  |  |  |  | |
|  | Ageing |  |  |  |  |  | |
|  | Accident or injury |  |  |  |  |  | |
|  | Manual work |  |  |  |  |  | |
|  | Sport |  |  |  |  |  | |
|  | Osteoarthritis |  |  |  |  |  | |
|  | Changes consistent with osteoarthritis seen on x-ray |  |  |  |  |  | |

| **We are interested in your own personal views of how you see chronic joint problems**.  Please indicate how much you agree or disagree with the following statements about chronic joint problems by putting a cross in one box on each line. | | | | | | | |  |
| --- | --- | --- | --- | --- | --- | --- | --- | --- |
|  |  | **Totally disagree** | **Largely disagree** | **Disagree to some extent** | **Agree to some extent** | **Largely agree** | **Totally agree** | |
|  | Mental stress can cause chronic joint problems even in the absence of tissue damage |  |  |  |  |  |  | |
|  | The cause of chronic joint problems is unknown |  |  |  |  |  |  | |
|  | Pain is a nociceptive stimulus, indicating tissue damage |  |  |  |  |  |  | |
|  | A patient suffering from a severe chronic joint problem will benefit from physical exercise |  |  |  |  |  |  | |
|  | Functional limitations associated with chronic joint problems are the result of psychosocial factors |  |  |  |  |  |  | |
|  | Patients with chronic joint problems should preferably practice only pain free movements |  |  |  |  |  |  | |
|  | Treatment may have been successful even if pain remains |  |  |  |  |  |  | |
|  | Chronic joint problems indicates the presence of organic injury |  |  |  |  |  |  | |
|  | If chronic join problem increases in severity, I immediately adjust the intensity of my treatment accordingly |  |  |  |  |  |  | |
|  | If treatment does not result in a reduction in chronic joint problem, there is a high risk of severe restrictions in the long term |  |  |  |  |  |  | |
|  | Pain reduction is a precondition for the restoration of normal functioning |  |  |  |  |  |  | |
|  | Increased pain indicates new tissue damage or the spread of existing damage |  |  |  |  |  |  | |
|  | Even if the pain has worsened, the intensity of the next treatment can be increased |  |  |  |  |  |  | |
|  | There is no effective treatment to eliminate chronic joint problems |  |  |  |  |  |  | |
|  | If patients complain of pain during exercise, I worry that damage is being caused |  |  |  |  |  |  | |
|  | The severity of tissue damage determines the level of pain |  |  |  |  |  |  | |
|  | Learning to cope with stress promotes recovery from chronic joint problems |  |  |  |  |  |  | |
|  | Exercises that may be joint straining should not be avoided |  |  |  |  |  |  | |
|  | In the long run, patients with chronic joint problems have a higher risk of developing severe functional impairments |  |  |  |  |  |  | |

| **Section 3 – Clinical scenario of patient with a chronic joint problem** | | | | | | | | | | | | | | | | | | | | | | | | | | | | | | |  | | | | | |
| --- | --- | --- | --- | --- | --- | --- | --- | --- | --- | --- | --- | --- | --- | --- | --- | --- | --- | --- | --- | --- | --- | --- | --- | --- | --- | --- | --- | --- | --- | --- | --- | --- | --- | --- | --- | --- |
| Presented below is a scenario of a patient **with a chronic joint problem** who has been to see their GP and has now been referred to you. All questions that follow relate to the care you would give this particular patient. Think about the patient’s **first consultation** with you. | | | | | | | | | | | | | | | | | | | | | | | | | | | | | | |  | | | | | |
| **Patient** | | | | | | | | | | | Mrs Jones, 58-year-old Prison Officer | | | | | | | | | | | | | | | | | | | |  | | | | | |
| **History** | | | | | | | | | | | First presentation of gradually worsening bilateral knee pain over 2 years  No history of trauma  Pain always present when walking and after sitting, worst when climbing stairs  No night pain.  Managing activities of daily living. Difficulty gardening.  Stopped going to gym – thinks was making pain worse Only treatment tried is ibuprofen once or twice when pain “really bad” no benefit.  Came today finding work increasingly difficult due to the stairs Usually well – no co-morbidities | | | | | | | | | | | | | | | | | | | |  | | | | | |
| **Medication:** | | | | | | | | | | | Nil | | | | | | | | | | | | | | | | | | | |  | | | | | |
| **Examination** | | | | | | | | | | | Body Mass Index 33 Knees – no effusions. Joint tenderness upon palpation. Bilateral coarse crepitations.  Slightly reduced flexion of the right knee  Hips – no abnormality detected  GP has made a working diagnosis of osteoarthritis | | | | | | | | | | | | | | | | | | | |  | | | | | |
| 3.1 | | | | | This patient’s symptoms are*: (Please cross* ***one*** *box that best reflects your opinion)* | | | | | | | | | | | | | | | | | | | | | | | | | |  | | | | | |
|  | | | | | □ | | | | Very severe | | | | □ | | Severe | | | | | | □ | Moderate | □ | Mild | | | □ | | | Very mild |  | | | | | |
|  | | | | |  | | | | | | | | | | | | | | | | | | | | | | | | | |  | | | | | |
| 3.2 | | | | | It is most likely that this patient’s symptoms result from joint damage that is:  *(Please cross* ***one*** *box that best reflects your opinion)* | | | | | | | | | | | | | | | | | | | | | | | | | |  | | | | | |
|  | | | | | □ | | | | Very severe | | | | □ | | Severe | | | | | | □ | Moderate | □ | Mild | | | □ | | | Very mild |  | | | | | |
|  | | | | |  | | | | | | | | | | | | | | | | | | | | | | | | | |  | | | | | |
|  | | | | |  | | | | | | | | | | | | | | | | | | | | | | | | | |  | | | | | |
| 3.3 | | | | | Using the words you would use with the patient, ***briefly*** state how would you describe the diagnosis that the doctor gave to the patient | | | | | | | | | | | | | | | | | | | | | | | | | |  | | | | | |
| …………………………………………………………………………………………………………  …………………………………………………………………………………………………………  …………………………………………………………………………………………………………  ……………………………………………………………………………………………………...... | | | | | | | | | | | | | | | | | | | | | | | | | | | | | | |  | | | | | |
| 3.4. | | | | | Using the words you would use with the patient, ***briefly*** describe what the future is likely to hold for this patient | | | | | | | | | | | | | | | | | | | | | | | | | |  | | | | | |
| …………………………………………………………………………………………………………  …………………………………………………………………………………………………………  ………………………………………………………………………………………………………… | | | | | | | | | | | | | | | | | | | | | | | | | | | | | | |  | | | | | |
| 3.5 | | | | **At this point**, what approaches would you use to treat this patient?  *(Please cross* ***all options*** *that apply)* | | | | | | | | | | | | | | | | | | | | | | | | | | |  | | | | | |
|  | | □ | | | | | Education, verbal advice | | | | | | | | | | □ | | | Assistive devices  (e.g. walking aids) | | | | | | □ | | | Paracetamol | | | | |  | | |
|  | |  | | | | |  | | | | | | | | | |  | | |  | | | | | |  | | |  | | | | |  | | |
|  | | □ | | | | | Strengthening exercises | | | | | | | | | | □ | | | Oral NSAIDS | | | | | | □ | | | Intra-articular steroid injection | | | | |  | | |
|  | | □ | | | | | Heat / ice | | | | | | | | | | □ | | | Rest | | | | | | □ | | | Opioids | | | | |  | | |
|  | |  | | | | |  | | | | | | | | | |  | | |  | | | | | |  | | |  | | | | |  | | |
|  | | □ | | | | | Provide written information | | | | | | | | | | □ | | | Topical NSAIDS | | | | | | □ | | | Use of joint support | | | | |  | | |
|  | |  | | | | | | | | | | | | | | | | | | | | | | | | | | | | | | | | | | |
|  | | □ | | | | | Capsacin | | | | | | | | | | □ | | | TENS | | | | | | □ | | | General physical activity | | | | |  | | |
|  | |  | | | | | | | | | | | | | | | | | | | | | | | | | | | | | | | | | | |
|  | | □ | | | | Avoidance of painful movement / activity | | | | | | | | | | | | □ | | | Shock absorbing shoes or insoles | | | | | □ | | | Reducing activity level | | | | |  | | |
|  | |  | | | |  | | | | | | | | | | | |  | | |  | | | | |  | | |  | | | | |  | | |
|  | | □ | | | | Weight loss | | | | | | | | | | | | □ | | | Pacing of activities | | | | | □ | | | Nutrition | | | | |  | | |
|  | |  | | | |  | | | | | | | | | | | |  | | |  | | | | |  | | |  | | | | |  | | |
|  | | □ | | | | Increasing activity level | | | | | | | | | | | | □ | | | Other If you crossed ‘other’, please specify | | | | | | | | | | | | |  | | |
|  | …………………………………………………………………………………………………………  ………………………………………………………………………………………………………… | | | | | | | | | | | | | | | | | | | | | | | | | | | | | | | | | | |  |
|  |  | | | | | | | | |  | | | | | | | | | | | | | | | | | | | | | | | | | |  |
| 3.6 | | | | | Would you be likely to **refer this patient** on to see someone else at this point? | | | | | | | | | | | | | | | | | | | | | | | | | |  | | | | | |
|  | | | □ | | | | | No | | | | □ | | Yes - If yes, to whom (please cross all options that apply) | | | | | | | | | | | | | | | | | | |  | | | |
|  | | |  | | | | |  | | | | | | | |  | | |  | | | | | |  | | |  | | | |  | | | | |
|  | | | □ | | | | | Orthopaedic surgeon | | | | | | | | □ | | | Occupational therapist | | | | | | □ | | | Pain clinic | | | |  | | | | |
|  | | | □ | | | | | Dietician | | | | | | | | □ | | | Local pharmacist | | | | | | □ | | | Acupuncturist | | | |  | | | | |
|  | | | □ | | | | | GP with a special interest | | | | | | | | □ | | | Rheumatologist | | | | | | □ | | | Physiotherapist | | | |  | | | | |
|  | | | □ | | | | | Podiatrist | | | | | | | | □ | | | Exercise on Prescription (or equivalent) | | | | | | □ | | | Support group | | | |  | | | | |
|  | | | □ | | | | | Other *(Please specify)*……………………………………………………………………....  ………............................................................................................................................ | | | | | | | | | | | | | | | | | | | | | | | | | | |  | |

| **Section 4 – About osteoarthritis** |
| --- |

|  | How well informed do you feel about the following aspects of osteoarthritis? **Please circle one response for each question in this section** | | | | | | | | | | |
| --- | --- | --- | --- | --- | --- | --- | --- | --- | --- | --- | --- |
|  | | |  | | | | | | | |  |
|  | | | What causes osteoarthritis | | | | | | | | |
|  | | Not at all informed | | |  | Partly informed | |  | Very well informed | | |
|  | | | **1** | **2** | | **3** | **4** | | **5** |  | |
|  | | |  |  | |  |  | |  |  | |
|  | | | The prognosis of osteoarthritis | | | | | | | | |
|  | | Not at all informed | | |  | Partly informed | |  | Very well informed | | |
|  | | | **1** | **2** | | **3** | **4** | | **5** |  | |
|  | | |  |  | |  |  | |  |  | |
|  | | | The burden (impact on daily life) of osteoarthritis on the individual | | | | | | | | |
|  | | Not at all informed | | |  | Partly informed | |  | Very well informed | | |
|  | | | **1** | **2** | | **3** | **4** | | **5** |  | |
|  | | |  |  | |  | |  |  |  | |
|  | | | The range of treatments for osteoarthritis | | | | | | | | |
|  | | Not at all informed | | |  | Partly informed | |  | Very well informed | | |
|  | | | **1** | **2** | | **3** | **4** | | **5** |  | |
|  | | |  |  | |  |  | |  |  | |
|  | | | What people with osteoarthritis can do to manage their condition | | | | | | | | |
|  | | Not at all informed | | |  | Partly informed | |  | Very well informed | | |
|  | | | **1** | **2** | | **3** | **4** | | **5** |  | |
|  | | |  |  | |  |  | |  |  | |
|  | | | What a nurses can do to support patients with osteoarthritis to self manage their condition | | | | | | | | |
|  | | Not at all informed | | |  | Partly informed | |  | Very well informed | | |
|  | | | **1** | **2** | | **3** | **4** | | **5** |  | |
|  | | |  |  | |  |  | |  |  | |

**Section 5 - The NICE Osteoarthritis Guideline and its recommendations**

**Please circle one response for each question in this section**

|  | How much have you heard or read about the NICE Osteoarthritis Guideline, published in 2008? | | | | | | | | |  | |
| --- | --- | --- | --- | --- | --- | --- | --- | --- | --- | --- | --- |
|  | Nothing at all |  | | Some | |  | | A lot | |  | |
|  | **1** | **2** | | **3** | | **4** | | **5** | |  | |
|  |  | | | | | | | | |  | |
|  | How much do you feel that NICE is a credible source of guidance for the management of osteoarthritis? | | | | | | | | |  | |
|  | Not at all |  | | Somewhat | |  | | A lot | |  | |
|  | **1** | **2** | | **3** | | **4** | | **5** | |  | |
|  |  | | | | | | | | |  | |
| The NICE Osteoarthritis Guideline made a number of recommendations. The next questions are about some of these recommendations | | | | | | | | | |  | |
|  |  | | | | | | | | |  | |
|  | How much have you heard or read about the recommendation that healthcare professionals should support patients with osteoarthritis to self-manage their condition? | | | | | | | | |  | |
|  | Nothing at all |  | | Some | |  | | A lot | |  | |
|  | **1** | **2** | | **3** | | **4** | | **5** | |  | |
|  |  | | | | | | | | |  | |
|  | 5.3.1. Do you agree with this recommendation? | | | | | | | | |  | |
|  | Completely disagree | |  | | Somewhat agree | |  | | Completely agree | | |
|  | **1** | | **2** | | **3** | | **4** | | **5** | | |
|  |  | | | | | | | | | |  |
|  |  | | | | | | | | | |  |
| . | 5.3.2. Do you provide support for patients with osteoarthritis to self-manage their condition? | | | | | | | | | |  |
|  | Never |  | | About half the time | |  | | Always | | |  |
|  | **1** | **2** | | **3** | | **4** | | **5** | | |  |
|  |  | | | | | | | | | |  |
| . | 5.3.3. If you have circled 3, 4, or 5 for the last question, how do you ensure that patients with osteoarthritis are supported to self-manage their condition? | | | | | | | | | |  |
|  | ………………………………………………………………………………………………… | | | | | | | | | |  |
|  | ………………………………………………………………………………………...........…. | | | | | | | | | |  |
|  | ………………………………………………………………………...........…………………… | | | | | | | | | |  |

| **Please circle one response for each question in this section** | | | | | | |
| --- | --- | --- | --- | --- | --- | --- |
| 5.4 | How much have you heard or read about the recommendation that healthcare professionals should offer all patients with osteoarthritis **written information** about their condition | | | | | |
|  | Nothing at all |  | Some |  | A lot |  |
|  | **1** | **2** | **3** | **4** | **5** |  |
|  |  | | | | |  |
| . | 5.4.1. Do you agree with this recommendation? | | | | |  |
|  | Completely disagree |  | Somewhat agree |  | Completely agree |  |
|  | **1** | **2** | **3** | **4** | **5** |  |
|  |  | | | | |  |
| . | 5.4.2. Do you provide **written information** for patients with osteoarthritis? | | | | |  |
|  | Never |  | About half the time |  | Always |  |
|  | **1** | **2** | **3** | **4** | **5** |  |
|  |  | | | | |  |
| . | 5.4.3. If you have circled 3, 4 or 5 for the last question, how do you **ensure** that patients with osteoarthritis are provided with **written information**? | | | | |  |
|  | ………………………………………………………………………………………...... | | | | |  |
|  | ………………………………………………………………………………………...... | | | | |  |
|  | ………………………………………………………………………………………...... | | | | |  |
|  | ………………………………………………………………………………………...... | | | | |  |
|  |  | | | | |  |
| . | 5.4.4. If you do provide written information can you provide details of the leaflets/ information you use – including website(s) if known | | | | |  |
|  | ………………………………………………………………………………………...... | | | | |  |
|  | ………………………………………………………………………………………...... | | | | |  |
|  | ………………………………………………………………………………………...... | | | | |  |
|  |  | | | | |  |

**Please circle one response for each question in this section**

|  |  | |  | | | | | | | | | |
| --- | --- | --- | --- | --- | --- | --- | --- | --- | --- | --- | --- | --- |
|  |  | | How much have you heard or read about the recommendation that healthcare professionals should offer **all** patients with osteoarthritis advice on **exercise and increasing physical activity?** | | | | | | | | | |
|  |  | | Not at all | |  | | Somewhat | |  | | A lot | |
|  |  | | **1** | | **2** | | **3** | | **4** | | **5** | |
|  |  | |  | | | | | | | | | |
|  | . | | 5.5.1. Do you agree with this recommendation? | | | | | | | | | |
|  | | Completely disagree | |  | | Somewhat agree | |  | | Completely agree | |  |
|  |  | | **1** | | **2** | | **3** | | **4** | | **5** | |
|  |  | |  | | | | | | | | | |
|  | . | | 5.5.2. Do you offer advice on exercise and increasing physical activity to patients with osteoarthritis? | | | | | | | | | |
|  |  | | Never | |  | | About half the  time | |  | | Always | |
|  |  | | **1** | | **2** | | **3** | | **4** | | **5** | |
|  |  | |  | | | | | | | | | |
|  | . | | 5.5.3. If you have circled 3, 4 or 5 for the last question, how do you **ensure** that patients with osteoarthritis are offered advice to undertake exercise or increase physical activity? | | | | | | | | | |
|  |  | | ………………………………………………………………………………………...... | | | | | | | | | |
|  |  | | ………………………………………………………………………………………...... | | | | | | | | | |
|  |  | | ………………………………………………………………………………………...... | | | | | | | | | |
|  |  | | ………………………………………………………………………………………...... | | | | | | | | | |
|  | . | | 5.5.4. Please list what exercises and ways to increase physical activity you offer advice on? | | | | | | | | | |
|  |  | | ………………………………………………………………………………………...... | | | | | | | | | |
|  |  | | ………………………………………………………………………………………...... | | | | | | | | | |
|  |  | | ………………………………………………………………………………………...... | | | | | | | | | |
|  |  | | ………………………………………………………………………………………...... | | | | | | | | | |
|  |  | |  | | | | | | | | | |

**Please circle one response for each question in this section**

|  |  | |  | | | | | | | | | |
| --- | --- | --- | --- | --- | --- | --- | --- | --- | --- | --- | --- | --- |
|  |  | | How much have you heard or read about the recommendation that healthcare professionals should offer **all** patients with osteoarthritis, if they are overweight or obese, advice on interventions to achieve **weight loss**? | | | | | | | | | |
|  |  | | Nothing at all | |  | | Some | |  | | A lot | |
|  |  | | **1** | | **2** | | **3** | | **4** | | **5** | |
|  |  | |  | | | | | | | | | |
|  |  | | 5.6.1. Do you agree with this recommendation? | | | | | | | | | |
|  | | Completely disagree | |  | | Somewhat agree | |  | | Completely agree | |  |
|  |  | | **1** | | **2** | | **3** | | **4** | | **5** | |
|  |  | |  | | | | | | | | | |
|  |  | | 5.6.2. Do you offer advice on interventions to achieve weight loss to patients with osteoarthritis, if they are overweight or obese? | | | | | | | | | |
|  |  | | Never | |  | | About half the time | |  | | Always | |
|  |  | | **1** | | **2** | | **3** | | **4** | | **5** | |
|  |  | |  | | | | | | | | | |
|  |  | | 5.6.3. If you have circled 3, 4 or 5 for the last question, how do you **ensure** that patients with osteoarthritis are offered advice on interventions to achieve **weight loss**, if they are overweight or obese? | | | | | | | | | |
|  |  | | ………………………………………………………………………………………...... | | | | | | | | | |
|  |  | | ………………………………………………………………………………………...... | | | | | | | | | |
|  |  | | ………………………………………………………………………………………...... | | | | | | | | | |
|  |  | | ………………………………………………………………………………………...... | | | | | | | | | |
|  |  | | 5.6.4. Please list which interventions to achieve weight loss you offer advice on: | | | | | | | | | |
|  |  | | ………………………………………………………………………………………...... | | | | | | | | | |
|  |  | | ………………………………………………………………………………………...... | | | | | | | | | |
|  |  | | ………………………………………………………………………………………...... | | | | | | | | | |
|  |  | | ………………………………………………………………………………………...... | | | | | | | | | |
|  |  | |  | | | | | | | | | |

Section 6 – Managing osteoarthritis in practice

**Please circle one response for each question in this section**

|  | How much do you feel it is part of a practice nurse / nurse practitioner’s job to manage people with osteoarthritis? | | | | | | |
| --- | --- | --- | --- | --- | --- | --- | --- |
|  | Not at all |  | | Somewhat |  | | A lot |
|  | **1** | **2** | | **3** | **4** | | **5** |
|  |  | | | | | | |
|  | How much is managing patients with osteoarthritis a priority for you? | | | | | | |
|  | Not a priority |  | | A medium priority |  | | A high priority |
|  | **1** | **2** | | **3** | **4** | | **5** |
|  |  | | | | | | |
| **For the next two questions please think about how you manage osteoarthritis in the consultation** | | | | | | | |
|  |  | | | | | | |
|  | Do you have enough time to manage osteoarthritis when it is the only problem being managed? | | | | | | |
|  | Not enough time |  | | Just enough time |  | | Plenty of time |
|  | **1** | **2** | | **3** | **4** | | **5** |
|  |  | | | | | | |
|  | Do you have enough time to manage osteoarthritis when there are other problems which also need to be managed? | | | | | | |
|  | Not enough time |  | | Just enough time |  | | Plenty of time |
|  | **1** | **2** | | **3** | **4** | | **5** |
|  |  | | | | | | |
|  | Do you feel confident about diagnosing osteoarthritis clinically (without the use of x-rays)? | | | | | | |
|  | Not confident |  | | Somewhat confident |  | | Very confident |
|  | **1** | **2** | | **3** | **4** | | **5** |
|  |  | | | | | | |
|  | Do you feel confident about examining peripheral joints in older patients? | | | | | | |
|  | Not confident |  | | Somewhat confident |  | | Very confident |
|  | **1** | **2** | | **3** | **4** | | **5** |
|  |  | | | | | | |
|  | Do you feel confident in prescribing medication for osteoarthritis? | | | | | | |
|  | Not confident |  | | Somewhat confident |  | | Very confident |
|  | **1** | **2** | | **3** | **4** | | **5** |
|  |  | | | | | | |
| **Please circle one response for each question in this section** | | | | | | | |
|  | Do you feel confident about supporting patients with osteoarthritis to self-manage their condition? | | | | | | |
|  | Not confident |  | | Somewhat confident |  | | Very confident |
|  | **1** | **2** | | **3** | **4** | | **5** |
|  | | | | | | | |
|  | How much do you think written information for patients with osteoarthritis helps them to better manage their condition? | | | | | | |
|  | Not at all |  | | Somewhat |  | | A lot |
|  | **1** | **2** | | **3** | **4** | | **5** |
|  |  | | | | | | |
|  | How much do you think exercise and increasing physical activity by people with osteoarthritis will improve their pain? | | | | | | |
|  | Not at all |  | | Somewhat |  | | A lot |
|  | **1** | **2** | | **3** | **4** | | **5** |
|  |  | | | | | | |
|  | How much do you think losing weight by people with osteoarthritis, if they are overweight or obese, will improve their pain? | | | | | | |
|  | Not at all |  | | Somewhat |  | | A lot |
|  | **1** | **2** | | **3** | **4** | | **5** |
|  |  | | | | | | |
|  | When wanting to refer a patient with osteoarthritis, do you have good access to physiotherapy services? | | | | | | |
|  | Very poor access |  | | Reasonable access |  | | Very good access |
|  | **1** | **2** | | **3** | **4** | | **5** |
|  |  | | | | | | |
|  | When wanting to refer a patient with osteoarthritis, do you have good access to occupational therapy services? | | | | | | |
|  | Very poor access |  | | Reasonable access |  | | Very good access |
|  | **1** | **2** | | **3** | **4** | | **5** |
|  |  | | | | | | |
|  | When wanting to refer a patient with osteoarthritis, do you have good access to rheumatology services? | | | | | | |
|  | Very poor access |  | | Reasonable access |  | | Very good access |
|  | **1** | **2** | | **3** | **4** | | **5** |
|  |  | | | | | | |
| **Please circle one response for each question in this section** | | | | | | | |
|  | When wanting to refer a patient with osteoarthritis, do you have good access to orthopaedic services? | | | | | | |
|  | Very poor access |  | | Reasonable access |  | | Very good access |
|  | **1** | **2** | | **3** | **4** | | **5** |
|  |  | | | | | | |
|  | How much do you have a "heart-sink" reaction to patients with osteoarthritis? | | | | | | |
|  | Not at all | | Somewhat | | | A lot | |
|  | **1** | **2** | | **3** | **4** | | **5** |

**Thank you**

**Now please read and, if you are happy to, initial and sign the consent form on the next page and return the questionnaire in the stamped addressed envelope.**

Study ID

Study ID
