# Supplementary material for: A nurse-led clinic for patients consulting with osteoarthritis in general practice: development and impact of training in a cluster randomised controlled trial
Source: BMC Fam Pract. 2016 Dec 21;17:173. doi: 10.1186/s12875-016-0568-y (PMC5178095; doi:10.1186/s12875-016-0568-y)
Supplement: Additional file 2: — Final practice nurse training outline. (DOCX 25 kb) [file 12875_2016_568_MOESM2_ESM.docx]

# Appendix 2: Practice nurse Final Training Programme

**Day 1 AM and PM: Introduction and communication sessions**

| **Practice Nurse training Day 1** | | |
| --- | --- | --- |
| **Learning outcomes**  **Knowledge of: NICE OA Guideline recommendations (especially on information, exercise, weight loss and simple analgesia)the content and style of the model OA consultation (GP consultation and nurse-led OA clinic), the principles of support for self-management, the structure of the training and its learning objectives, effective communication in the consultation, the case report form, the resource folder**  **Attitudes: feel positive about adopting a patient-centred approach in the nurse-led OA clinic , appreciate the significance of psychosocial issues when tailoring NICE OA recommendations to individual patients**  **Skills: adopting a patient-centred approach in the consultation, the nurse-led OA clinic consultation skills (introductions, GP consultation debrief and use of the CRF, use of OA Guidebook, agenda, giving explanations on OA and its treatment, provide self-management support** | | |
| **TIME** | **CONTENT** | **RESOURCES - notes** |
| 9.30 – 10.00 | All introduce themselves with a brief description from the practice nurses / healthcare support workers of their role in the practice |  |
| 10.00 – 10.30 | Recap on the combined GP and nurse training session 1. Then discussion and identify any learning needs which need to be addressed in the practice nurse training | 5 slide presentation recapping combined GP and nurse training session 1 - focussing on what we are trying to implement in 10 care: support for self-management and NICE OA core treatments  Flip chart to capture discussion and learning needs |
| 10.30 – 11.00 | The WHAT of the nurse-led OA clinic.  Recap on the GP consultation.  Then discussion on what we want to achieve over the four nurse-led OA clinic appts. | 1 slide presentation to recap on GP key tasks – the nurse-led OA clinic starts where the GP consultation leaves off  Flip chart to capture key points - of the WHAT and the HOW  THE WHAT – the outcome for patients (where do we want patients to get to at the end of the nurse-led OA clinic)  Trainers to prompt for: patient understands OA and its treatment, gained increased confidence and skills, more motivated to self-manage, if appropriate addressing weight, exercises and physical activity, and pain management |
| 11.00 – 11.10 | BREAK |  |
| 11.10 – 12.10 | The having got out the WHAT go through the HOW of the nurse-led OA clinic in the same way  AGREE outline and style of the nurse-led OA clinic  How we are going to get the nurses proficient in delivering the nurse-led OA clinic.  Go over areas of expertise of the nurses: what they know already of the WHAT and HOW  Go over the practice nurse training programme | THE HOW – how do we get the patients there  Trainers to prompt for: understanding patient story, how life affected, level of pain, mobility and activities, what tried, what happened when they saw the GP, ideas about OA, understanding of OA, OA explanation giving,  PATIENT-CENTRED APPROACH (slide)  (take a biopsychosocial approach, treat the patient as a person, bring your humanity to the consultation, share power and responsibility, forge a therapeutic alliance)  THE BEVIOURAL APPROACH to increase patient confidence and skills, agenda setting, goal setting, identifying obstacles, SMART, assessing confidence and importance, problem solving, using diaries, joint examination,  Using the OA Guidebook  Providing advice on pain, exercise and physical activity  4 slides outlining practice nurse training – the first consultation, SMART, joint examination, study procedures, addressing exercise, weight and pain, SPs, putting it all together, evaluation |
| 12.10 – 12.15 | COMFORT BREAK |  |
| 12.15 – 12.45 | Watch MP/SP video of GP consultation (BM video)  Flow of 1^st^ consultation  Introduce the case report form | Facilitated discussion of consultation  Flip chart and prompt for - introduction, understanding the story (pain, function, activities, how life affected, how copes, what tried), what happened in the GP consultation (the four questions on the CRF), OA explanations and use of the OA Guidebook, treatments for OA, agenda setting  The first four questions before giving explanations |
| 12.45 – 13.00 | LUNCH |  |
| 13.00 – 14.00 | External seminar |  |
| 14.00 - 14.30 | Communication in the consult. | Brief presentation from CM on communication, beliefs |
| 14.30 - 14.35 | BREAK |  |
| 14.35 - 15.45 | Use of CRF  Role play of 1^st^ consultation | Introductions, explaining OA and its treatments, use of CRF and OA Guidebook – trio work (observers to report on use of jargon / clarity / conciseness) - feedback at end |
| 15.45 - 16.00 | Wrap up session and preparation for day 2  Hand out resource folder  Homework – look at SMART tool, joint exam quiz, give out AR-UK exercise sheet to familiarise themselves with | Needs supplies of ARUK exercise sheet / booklet |

**Day 2 AM: GOAL SETTING**

| **Practice Nurse training Day 2** | | |
| --- | --- | --- |
| **Learning outcomes**  **Knowledge of : the SMART tool**  **Attitudes:**  **Skills: use of the SMART tool in negotiating goals for exercise, weight and pain management** | | |
| **Date and time** | **Content** | **Resources - notes** |
| 9:30- 1045 am | Presentation slides: To understand the purpose of goal setting | Powerpoint, discussion and exercises on own goals |
| 10:45-11:00 | **Break** |  |
| 11:00 – 12:15 | Use of Case Studies:  Goal setting using the SMART approach covering:   - Weight - Exercise - Medication - Pacing (if time)   Use of Case Report and SMART tool | SM to lead with other facilitators: Group work/ Role Play |

**Day 2 PM: living with OA, joint examination, study procedures**

| **Practice Nurse training Day 2** | | |
| --- | --- | --- |
| **Learning outcomes**  **Knowledge of: MOSAIC study procedures and reporting procedures, the signs osteoarthritis in a joint, the experience of a patient presenting with OA, the normative professional discourse in relation to the ways older patients perceive and manage their health and wellbeing, the complex and contingent nature of patient decision making, self-management from a patient perspective**  **Attitudes: use the understanding of the patient experience of OA when consulting for OA**  **Skills: Be able to handle painful joints and palpate keys features** | | |
| **Date and time** | **Content** | **Resources – notes** |
| 12.45 – 14.40 | Living with OA | Discussion and use of patient stories |
| 14.40 – 14.50 | **Break** |  |
| 14.50 – 15.15 | Joint examination | Plastic joint, (from AF), a plinth, PowerPoint  Flipchart and pens |
| 15.15 – 15.55 | Study procedures | Power point and flipcharts and CRF as homework  Four case studies to allow them to stimulate the recording of a consultation with each of these cases. |
| 15.55 – 16.00 | Homework for day 3: to re-listen to tracks 1,2,3 and 5 of Chronic Pain CD, come in clothing for practising exercises | Spare copies of Chronic Pain CD |

**Day 3 AM: Exercise**

| **Practice Nurse training Day 3** | | |
| --- | --- | --- |
| **Learning outcomes**  **Knowledge of: the benefits, and safety of exercise and activity for patients with OA**  **Attitudes:**  **Skills: confidence in explaining how exercise works in ways that patients will understand, confidence and competence in: i) facilitating patients to exercise and to be more active in the presence of pain, ii) prescribing, supervising and reviewing basic strengthening exercises, iii) facilitating patients to engage in and maintain more physically active lifestyles** | | |
| **Date and time** | **Content** | **Resources - notes** |
| 9:30- 10.00 am |  |  |
| 10.00 -11.10 | Part A (20 mins): Theory based  - Effects of exercise/ physical activity (PA)  - Safety of exercise/ PA in presence of co-morbidities  - Exercise/ PA in the presence of pain  Part B (50 mins): Prescribing local joint exercises for OA  - Teaching basic exercise techniques  - Review of exercises  - Simulation of initial prescription, setting exercise SMART goals and review of exercises | Hands on exercise AR-UK sheets  1 – 3 plinths  Presentation  SMART goal setting sheets  Nurses in clothing suitable for practising exercises |
| 11.10 – 11.20 | **Break** |  |
| 11:20 – 12.15 | Part C (55 mins): Physical activity for OA  - Group discussion around type and frequency intensity of PA and overcoming barriers to physical activity  - Use of pedometers  Practical demonstration/ participation in ‘moderate intensity’ PA  - Simulation of advising a patient to increase PA and setting SMART PA goals  - Simulation of a review of PA (if time)  - Incorporating exercise and PA together as 1 intervention for patients  HOMEWORK SETTING: Complete exercises and use of pedometer as you have been advised in this session until training day 4 | SMART goal sheets  Pedometers for each trainee  Hand out on co-morbidities (based on pilot training)  Article for further reading: Roddy et al 2005 MOVE exercise recommendations for hip and knee OA |

**Day 3: PM: Pain Management & Weight Management**

| **Practice Nurse training Day 3** | | |
| --- | --- | --- |
| **Learning outcomes**  **Knowledge of: NICE OA Guideline recommendations (especially on simple analgesia and interventions in the outer ring), pain mechanisms (chronic pain / OA pain), effective interventions for weight loss, how to help patients overcome obstacles to behaviour change**  **Attitudes: Understand and implement strategies to blend a patient-centred approach with guidelines and specific techniques; develop understanding and ownership of the “toolkit” items**  **Skills:, The nurse-led OA clinic consultation skills to address common medication issues in the consultation, motivational interviewing techniques for addressing weight loss** | | |
| **Date and time** | **Content** | **Resources - notes** |
| 12:45-13:15 | Pain Mechanisms | Powerpoint and discussion: CM |
| 13:15-14:15 | Pain Medication Management What can you do about pain?   - Tips for paracetamol and topical NSAIDs (15mins) - Role play of case scenarios – addressing medication abdication, compliance , side-effects | Discussion led by MP and SR  Each case scenario has first and second consultation (GM to lead) |
| 14:15:14:25 | **BREAK** |  |
| 14:25:14:40 | Weight management:   - how to work with patients to lose weight - effective treatments for weight loss - how are we going to use expertise of practice nurses in consultation approach | MP to lead with practice nurses  Hand-out about weight management;  Motivational Interviewing |
| 14:40: 14:55 | NICE outer ring treatments: Structured discussion on use | MP to lead ; one slide on NICE treatment recommendations and cross referencing to OA guidebook |
| 14:55 -15:55 | Overcoming obstacles to behaviour change  Discussion about the subsequent consultations  How they are going to be addressed in the consultation: 1) behavioural techniques; 2) skills and motivation to do it. l  Outcome: list of techniques to try with simulated patients on day 4: Around: Medication use, weight loss; and exercise. | CM to lead |

**Day 4**

| **Practice Nurse training Day 4** | | | |
| --- | --- | --- | --- |
| **Learning outcomes**  **Attitudes: Elicit, value and work with the patient’s story, understanding and health beliefs to devise a collaborative action plan towards a shared goal**  **Skills: Practising and refining consultations skills; practising study procedures and reporting** | | | |
| **Time** | **Activity** | **Resources** | **KCF** |
| 09.30– 10.00 | Introduction to working with simulated patients  Plan which part of consultation to work on:  First nurse consultation  Use of CRF (first section)  Explaining OA  Agenda setting | VC, SR, MP, CM |  |
| 10.00-11.00 | Working with SP as above | SP role F, VC, SR, MP, CM |  |
| 11.00-11.15 | Break |  |  |
| 11.15-12.15 | Continuing SP work on first nurse consultation:  Agreeing action plan  Goal setting | As above |  |
| 12.15-12.45 | **Lunch** |  |  |
| **Practice Nurse training Day 4** | | | |
| **Learning outcomes**  **Knowledge: Understand patient resistance and techniques for achieving outcomes**  **Attitudes: Elicit, value and work with the patient’s story, understanding and health beliefs to devise a collaborative action plan towards a shared goal**  **Skills: Develop confidence in advising on and demonstrating exercises. Develop consultation skills to include specific behaviour change techniques** | | | |
| **Time** | **Activity** | **Resources** | **KCF** |
| 12.45-13.30 | a) Feedback and learning from their own experiences of following an exercise and PA programme  b) Revision and further skills development – specific exercises | MH  3 plinths |  |
| 13.30-13.45 | Planning simulated patient exercise:  Follow-up nurse consultations  Working with resistance | VC, SR, MP, CM |  |
| 13.45-14.45 | Working with SP as above | SP role B  VC, SR, MP, CM |  |
| 14.45-15.15 | Wrap up and action planning including use of the template for recording MOAC-2 activity | SR, CM, KD, MP |  |
| 15.15-16.00 | NPT interview – discussion about the training programme | PO, LB |  |
