# Supplementary material for: A nurse-led clinic for patients consulting with osteoarthritis in general practice: development and impact of training in a cluster randomised controlled trial
Source: BMC Fam Pract. 2016 Dec 21;17:173. doi: 10.1186/s12875-016-0568-y (PMC5178095; doi:10.1186/s12875-016-0568-y)
Supplement: Additional file 3: — The different patient scenarios used in the training. (DOCX 11 kb) [file 12875_2016_568_MOESM3_ESM.docx]

**Appendix 3: The different patient scenarios used in the training.**

**Scenarios A (knee) and B (hip)**

A patient with ischaemic heart disease, who had tried simple analgesia and thought their joint problem was due to “wear and tear”. They had concerns about exercise for example, that exercise was not safe and that it was difficult to exercise locally.

**Scenarios C (knee) and D (hip)**

A patient with diabetes who had tried over the counter painkillers and was concerned they had rheumatoid arthritis. They were overweight and had tried to lose weight many times before and had not succeeded.

**Scenarios E (knee) and F (hip)**

A patient with hypothyroidism who only occasionally took painkillers and thought they had arthritis as they are getting older. They had concerns about taking tablets which they thought were addictive and often give them side effects.
